# Extracellular matrix guidance of autophagy: a mechanism regulating cancer growth

**DOI:** 10.1098/rsob.210304

**Published:** 2022-01-05

**Authors:** Carolyn G. Chen, Renato V. Iozzo

**Affiliations:** Department of Pathology, Anatomy and Cell Biology and the Translational Cellular Oncology Program, Sidney Kimmel Cancer Center, Sidney Kimmel Medical College at Thomas Jefferson University, Philadelphia, PA 19107, USA

**Keywords:** proteoglycan, decorin, perlecan, collagen VI, angiogenesis

## Abstract

The extracellular matrix (ECM) exists as a dynamic network of biophysical and biochemical factors that maintain tissue homeostasis. Given its sensitivity to changes in the intra- and extracellular space, the plasticity of the ECM can be pathological in driving disease through aberrant matrix remodelling. In particular, cancer uses the matrix for its proliferation, angiogenesis, cellular reprogramming and metastatic spread. An emerging field of matrix biology focuses on proteoglycans that regulate autophagy, an intracellular process that plays both critical and contextual roles in cancer. Here, we review the most prominent autophagic modulators from the matrix and the current understanding of the cellular pathways and signalling cascades that mechanistically drive their autophagic function. We then critically assess how their autophagic functions influence tumorigenesis, emphasizing the complexities and stage-dependent nature of this relationship in cancer. We highlight novel emerging data on immunoglobulin-containing and proline-rich receptor-1, heparanase and thrombospondin 1 in autophagy and cancer. Finally, we further discuss the pro- and anti-autophagic modulators originating from the ECM, as well as how these proteoglycans and other matrix constituents specifically influence cancer progression.

## Graphical abstract

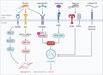

## 1. Introduction

The extracellular matrix (ECM) has long been understood as the dynamic macromolecular network that contributes both biophysical and biochemical factors to regulate tissue homeostasis. Through continuous remodelling, the ECM changes its composition, elasticity and structure to influence a wide array of biological functions, including adhesion, proliferation, wound healing, differentiation, migration and angiogenesis [1–4]. The ECM also has a profound impact on intracellular signalling as it oversees the storage and release of growth factors such as vascular endothelial growth factor (VEGF), fibroblast growth factor (FGF), epidermal growth factor (EGF), transforming growth factor β (TGFβ) and many others [5–7]. Given the extraordinary responsiveness of the ECM to physiological changes in the tissue, these processes that are intrinsic to the ECM allowing it to play a critical role in physiological settings can also be dysregulated in disease pathology [8].

In fibrosis and cancer, aberrant matrix remodelling and abnormal ECM deposition alter the makeup, rigidity and stiffness of the matrix [1,9,10]. Many cell types in the ECM, including fibroblasts, epithelial cells, immune cells and endothelial cells, deposit extracellular molecules and contribute to the elasticity and turnover of the matrix. Of these, fibroblasts are the predominant cells that synthesize and maintain the bulk of these ECM molecules that control the overall structure and mechanical properties of the matrix. For example, fibroblasts secrete elastin, collagens, glycosaminoglycans and glycoproteins, and can also differentiate into myofibroblasts under the influence of TGFβ and elevated tensile stress. Myofibroblast differentiation can then lead to increased deposition of fibrillar collagens and fibronectin, resulting in fibrotic changes and aberrant wound healing [11]. Fibroblasts also drive matrix proteolysis via matrix metalloproteinases (MMPs) and ADAMTS (a disintegrin and metalloproteinase with thrombospondin motifs) family proteases [12,13].

In cancer, solid tumours manipulate the ECM to establish and maintain a tumour-supportive milieu via secreting matrix components and enzymes that change the makeup of their microenvironment [14]. One common effect of matrix remodelling in the tumour milieu is desmoplasia, the increased deposition of fibrillar collagen and cross-linkage causing elevated tissue rigidity and stiffness [10]. Another oncological process perpetuated by the matrix is epithelial–mesenchymal transition (EMT), the process by which epithelial cell-derived carcinomas lose their tight cell–cell contacts, change morphology and motility, and metastasize from the tumour mass [15]. Multiple matrix molecules oversee EMT, including TGFβ [10]. Concurrently, cancer cells modify the activity of stromal cells to potentiate oncogenesis through matrix remodelling. Incited by biophysical changes in the ECM stiffness such as desmoplasia and continual paracrine exposure to neighbouring cancer cells, resident fibroblasts within a solid tumour differentiate into cancer-associated fibroblasts (CAFs) [16,16,17]. CAFs, in turn, recruit endothelial and immune cells to the tumour via secreting a variety of chemokines and growth factors including VEGFA (vascular endothelial growth factor A), PDGF (platelet-derived growth factor) and HGF (hepatocyte growth factor). Overall, activation of CAFs is integral in tumour vascularization, progression and chemoresistance [18–20], leading to unfavourable prognoses and clinical outcomes [21].

## 2. Effect of autophagy on tumour progression

The autophagic machinery functions as a counterpoint to cell growth and anabolic events activated when growth is not possible or is suppressed [22]. Therefore, there is an intrinsic antagonism between autophagy and growth. Macroautophagy (herein referred to as autophagy) is an intracellular process by which damaged or unused proteins, lipids and organelles in the cytoplasm undergo catalysis via the autophagosome-lysosomal pathway [23]. It involves approximately 20 autophagy-related (ATG) proteins that initiate and perpetuate autophagy, starting from the pre-autophagosomal structure (PAS) maturing into a double-membrane, spherical autophagosome to the final step of lysosomal fusion [24–26]. Self-digestion through this conserved pathway allows for the recycling of nucleotides, fatty acids, amino acids and sugars to maintain intracellular homeostasis [23,27]. Although the precise influence of autophagy on carcinogenesis has been widely debated, autophagy is generally understood to inhibit tumour growth and invasion in the early stages of tumour growth while promoting tumour invasion and metastasis in later stages of tumorigenesis.

In the early stages, autophagy inhibits mutagenesis, chronic inflammation and tissue injury, processes that collectively foster tumorigenesis [28–30]. For instance, enhanced autophagy blocks Human Epidermal Growth Factor 2 (HER2)-driven mammary tumorigenesis suggesting that therapies directed at increasing autophagic flux may become an appropriate therapeutic strategy for HER2-positive mammary breast cancers [31]. Autophagy also suppresses tumorigenic build-up of p62 [32] and deletion of critical autophagy genes *Atg7* and *Becn1*, resulting in malignancy in mice [33–35]. By contrast, later stages of oncogenesis are supported by autophagic activation, in which autophagy promotes tumour invasion and spread, growth, survival and chemotherapy resistance. It does this via maintaining tumour cell viability and homeostasis, conferring cytoprotection against metabolic stress and nutrient deprivation, DNA damage and hypoxia [28,30,36–39]. More recently, a developing and evolving body of work investigates the autophagic regulatory cues that originate from proteoglycans in the ECM. These autophagic modulators, both pro- and anti-autophagic in nature, are now being explored within the realm of the tumour microenvironment. Their implications on oncogenesis are critical in understanding the impact of the ECM on tumour progression, especially in the context of tumour vascularization.

## 3. Pro-autophagic signalling

**Figure 1.**
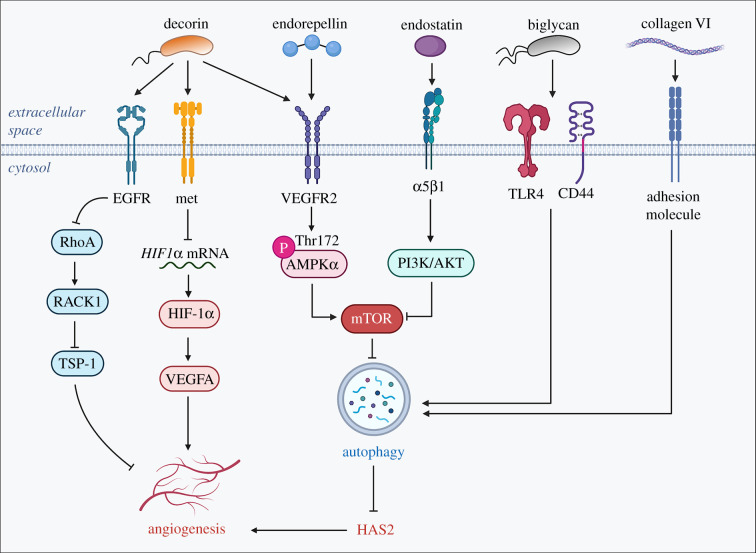
Schematic depiction of various pro-autophagic ECM components, their respective receptors and downstream signalling events. Please consult the text for additional information. The graphic was created with BioRender.com.

An emerging field of research that was overlooked for many years is based on the discovery that a soluble proteoglycan named decorin is capable of inducing autophagic flux in endothelial cells of various histogenetic backgrounds [40] (figure 1). After this original discovery made nearly 10 years ago, other proteoglycans and bioactive fragments have emerged as pro- or anti-autophagic molecules [41–43]. One example is endorepellin, the C-terminal domain V of the large multi-domain heparan sulfate proteoglycan (HSPG) perlecan [44–46], which is liberated from the endothelial basement membrane as a bioactive, processed species [47–49]. Originally, perlecan was isolated from renal basement membranes [50] but was later discovered to be a constituent of the colon carcinoma cell surface [51] and was eventually found in nearly all cells and tissues, both vascular and avascular [52,53]. Perlecan was soon identified as a major driver of angiogenesis via binding to angiogenic growth factors such as FGF2 and using antisense approaches in various systems [54–56].

Signalling through its 3 laminin-like globular (LG1-3) domains [57], endorepellin exerts pro-autophagic and angiostatic effects on vascular endothelia [58–60] by using a complex dual receptor antagonism, whereby concurrently interacts with VEGF receptor 2 (VEGFR2) and α2β1 integrin [61], two transmembrane receptors that are uniquely co-expressed in vascular endothelial cells [62–64]. Through its LG1/2 domains binding to the VEGFR2 immunoglobulin (Ig) 3–5 ectodomain motifs [65,66], endorepellin signals as partial agonist to activate autophagic machinery and suppress angiogenesis downstream [67–70]. Specifically, endorepellin activates VEGFR2 signalling, leading to downstream phosphorylation and activation of adenosine monophosphate (AMP)-activated protein kinase (AMPK) at Thr172 on its α subunit. This results in the canonical inhibition of mTOR [67], upregulation of autophagy proteins Peg3, LC3-II, Beclin 1 and p62 and formation of autophagosomes containing LC3, Peg3, p62, Beclin 1, Vps34 and mTOR [67,68]. Recently, we also discovered that endorepellin-evoked autophagy results in catabolic degradation of hyaluronan synthase 2 (HAS2), leading to a significant reduction in extracellular hyaluronan (HA) and angiogenic inhibition [69,70]. Notably, interfering with HA metabolism suppresses glioma cell proliferation by regulating autophagy [71].

In cancer, endorepellin demonstrates promising anti-oncogenic potential by inhibiting tumour growth, neovascularization and metabolism in squamous carcinoma and Lewis lung carcinoma [3,72]. Interestingly, endorepellin has also shown promise as a biomarker as systemic levels of its LG3 domain were elevated *in vivo* in pancreatic and breast cancer as well as a variety of other diseases including chronic renal nephropathy, IgA nephropathy, premature rupture of fetal membranes, Down syndrome and refractory cytopenia with multilineage dysplasia [73–83].

Another bioactive proteoglycan fragment, endostatin, is the N-terminal domain of the HSPG collagen XVIII and exerts pro-autophagic and anti-angiogenic effects in the vascular endothelial basement membrane [84]. Signalling through α5β1 integrin, endostatin activates autophagy in vascular endothelia [85] while concurrently inhibiting cell migration [86]. Like endorepellin, endostatin also induces autophagic degradation of HAS2, which would also inhibit HA-induced angiogenesis downstream [69,70]. In the context of cancer, endostatin also exhibits anti-cancer properties by therapeutically suppressing tumour vascularization in breast cancer and malignant keratinocytes [87]. Of note, Endostar, a novel recombinant human endostatin, effectively induces autophagy in human hepatocellular carcinoma cells, resulting in a marked suppression of tumour growth and increased cell death [88]. Furthermore, treatment of Endostar in murine lung carcinoma also suppressed tumour vascularization while concurrently activating autophagic machinery through the PI3 K/AKT/mTOR pathway [89].

Decorin, a small leucine-rich proteoglycan [57], is another well-studied molecule with a vast interacting network [90,91] and broad biological activities in various organs and specialized tissues including bone [92], tendon/ligaments [93–97], teeth and skin, among others. Soluble decorin acts as a monomer in solution [98] and binds to several receptor tyrosine kinase (RTK), including epidermal growth factor (EGFR) [99–101], Met [102] and insulin growth factor receptor-1 (IGFR-1) [103,104], thereby inducing autophagy via outside-in cues from the ECM [105]. Like endorepellin, decorin binds to VEGFR2, leading to phosphorylation of AMPK and upregulation and recruitment of LC3, Beclin 1 and Peg3 to the autophagosome [40,106–108]. Consequently, endothelial VEGFA is profoundly degraded downstream of decorin-evoked autophagy [109,110], while secretion of the angiostatic molecule thrombospondin-1 is upregulated extracellularly [111,112]. In the cancer milieu, decorin signals through EGFR and Met receptors found on the surface of cancer cells, effectively resulting in decreased HIF-1α and marked angiostasis of the tumour vasculature, suppression of tumour growth and enhanced mitophagy [99,102,110,110,113,114]. In response to fasting *in vivo*, decorin is also critical in overseeing the metabolic changes in the hexosamine biosynthetic pathway and modulations in cardiac function [115]. Notably, these initial observations of decorin-evoked autophagic flux have been recently confirmed in other systems including intervertebral disc cells [116], trophoblasts [117] and glioma cells [118]. Our recent discovery that decorin deficiency promotes EMT and colon cancer metastasis [119] is supported by the observations that exogenous decorin inhibits EMT in glioma cells [118] as well as inflammatory breast cancer growth and metastasis [120].

Another small leucine-rich proteoglycan, biglycan induces autophagosome formation in both macrophages and cardiomyocytes via signalling through the TLR4–CD44 receptor complex and TLR4 alone, respectively. In renal ischemia/reperfusion injury (IRI), biglycan not only recruits M1 macrophages to the site of renal IRI, but it also increases autophagy within these macrophages, effectively curtailing kidney inflammation and tubular damage at the renal ischemic site [121]. Indeed, biglycan is involved in a hepatorenal cross-talk, that is, biglycan produced by the cirrhotic liver could be a circulating messenger for renal pathophysiology via triggering inflammation and autophagy, ultimately affecting disease outcome [122]. In cardiomyocytes, biglycan confers cytoprotective mechanisms from IRI-induced tissue damage while modulating autophagy [123]. However, unlike decorin, endorepellin, endostatin and biglycan foster tumour angiogenesis in metastatic cancer via upregulating VEGFA levels and VEGFA–VEGFR2 signalling [124,125]. Tumour endothelia further exploit these pro-angiogenic effects by epigenetically activating *BGN* expression via hypomethylation of its promoter [126,127]. These pro-autophagic effects of biglycan have yet to be explored in the extracellular milieu of cancer. Thus, future investigations on the role of biglycan in modulating autophagy in tumour-associated macrophages and its effects on tumour angiogenesis would provide invaluable information on matrix-derived autophagic stimuli in cancer inflammatory response.

Collagen VI is an ECM protein secreted by fibroblasts in a wide variety of tissues that is deposited as a microfibrillar network in the matrix through a series of complex biosynthetic steps and filamentous assembly. In addition to maintaining biomechanical integrity, collagen VI offers cytoprotective roles in a broad spectrum of cell types, including chondrocytes, neurons, fibroblasts, cardiomyocytes and myofibres [128]. For instance, collagen VI inhibits spontaneous apoptosis and oxidative stress in central nervous system neurons [129]. Collagen VI is also essential in regulating autophagic flux, a critical component to establishing its cytoprotective functions [130]. In skeletal muscle, collagen VI-evoked autophagic flux in myocytes prevents myofibre degeneration due to collagen VI muscular dystrophies [131] or muscle wasting due to physical exercise [132]. Interestingly, activating autophagy via spermidine or pterostilbene both reduced muscle defects and myopathy in collagen VI-deficient mice [133,134].

In cancer, collagen VI promotes tumour angiogenesis and inflammation via the recruitment of endothelial cells and macrophages. Endotrophin, the C5 fragment of the α3 chain of collagen VI, functions as a chemoattractant for endothelial cell recruitment, thus inducing angiogenesis in the tumour microenvironment [135]. Finally, collagen VI further aids tumour progression by inducing EMT and promoting chemotherapy resistance [136]. While its pro-tumorigenic functions have not been investigated in the context of its pro-autophagic capabilities, this unexplored area of study would be highly anticipated as the role of ECM-derived autophagic regulators in cancer progression has been demonstrated in a number of matrix proteins.

VEGFA is a dimeric glycoprotein involved heavily in angiogenesis through the regulation of endothelial cell proliferation, survival and migration, as well as vascular permeability. A well-studied angiogenic factor in the matrix, VEGFA, is upregulated in hypoxic conditions via signalling through VEGFR2 [137]. Not only paramount in overseeing the angiogenesis, VEGFA also is itself a regulator of autophagy. In bovine ovarian granulosa cells, VEGFA induces autophagy through the AKT/PI3 K pathway such that siRNA knockdown of VEGFA inhibits autophagy and attenuates AKT phosphorylation, whereas VEGFA overexpression promotes autophagy and increases AKT phosphorylation [138]. In turn, autophagy also regulates VEGFA levels downstream, where VEGFA itself is an autophagic substrate in endothelial cells such that inhibiting autophagy results in an accumulation of intracellular VEGFA and activating autophagy depletes VEGFA levels [109]. Further, autophagic deficiency via *atg7* deletion in uterine stromal cells results in elevated VEGFA and increased vascular permeability [139].

VEGFA plays a key role in promoting tumour angiogenesis and is targeted in many anti-angiogenic therapies [137]. However, the specific functions of VEGFA-evoked autophagy in regulating cancer progression have yet to be investigated in depth. Of note, one study of ovarian cancer demonstrates that autophagy induced downstream of VEGFA promotes chemotherapy resistance, such that suppressing *VEGFA* expression inhibits autophagy biomarkers, increases apoptosis and decreases chemotherapeutic resistance overall [140]. Further investigation into the role of VEGFA on autophagy and cancer pathology would benefit this growing field of matrix-derived autophagic effectors in the context of cancer.

Although the molecular mechanism of several of the above-mentioned pro-autophagic inducers is not yet completely elucidated, the overwhelming majority of these extracellular ‘outside-in’ pro-autophagic cues [105] occur in the absence of nutrient deprivation and are thus considered non-canonical routes of autophagy induction. As the field of pro-autophagic ECM proteins in cancer expands, the breadth of understanding and utilization of these non-canonical forms of autophagic activation in treating cancer will also evolve.

## 4. IGPR-1, a novel bridge between cell adhesion and autophagy

**Figure 2.**
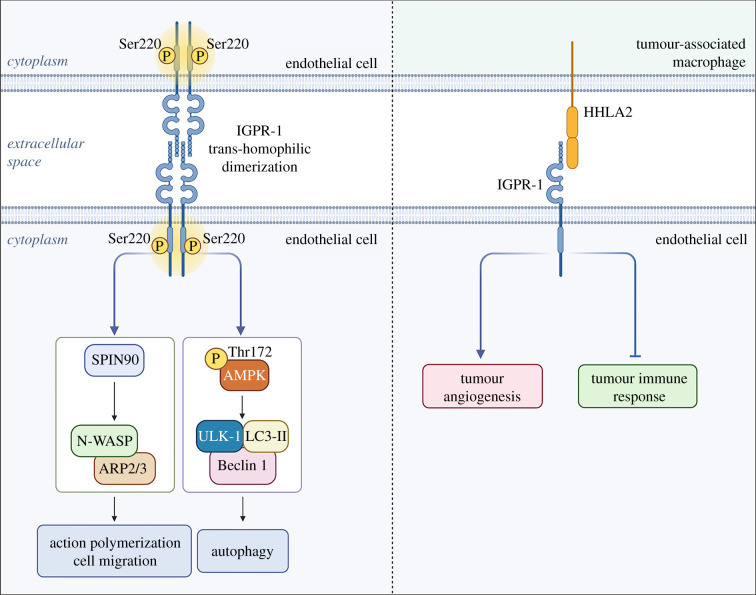
Schematic showing the functional roles of the cell adhesion molecule IGPR-1 in endothelial cells. At the endothelial-endothelial cell interface, IGPR-1 binds to itself via trans-homophilic dimerization, resulting in phosphorylation at Ser220 and autophagy and cell migration downstream. In the tumour microenvironment, IGPR-1 on the endothelial cell surface binds to HHLA2 on the tumour-associated macrophage, resulting in tumour angiogenesis and attenuated immune response. The graphic was created with BioRender.com.

There is mounting evidence for a cooperative exchange between cell adhesion and autophagy. Specifically, the autophagic flux can regulate adhesion dynamics to mediate neurite outgrowth and synaptic plasticity [141]. Immunoglobulin-containing and proline-rich receptor-1 (IGPR-1), also known as transmembrane and immunoglobulin domain containing 2 (TMIGD2), is a novel cell adhesion molecule expressed on the surfaces of epithelial and endothelial cells that has become a promising matrix protein of interest due to its unique role at the endothelial-cancer cell interface [142,143]. Although its signalling is not triggered by matrix-residing molecules, IGPR-1 receives extracellular signalling through cell–cell interactions, which propagates intracellular signalling cascades that modulate both autophagy and tumour angiogenesis (figure 2). In endothelial cells, IGPR-1 is localized in adherens junctions and undergoes trans-homophilic dimerization to maintain endothelial cell–cell adhesion and barrier function. Of note, the trans-homophilic dimerization of IGPR-1 between adjacent endothelial cells results in its phosphorylation at serine 220. This phosphorylation is necessary for its role in regulating cell–cell adhesion [144]. Like endorepellin and endostatin, IGPR-1 inhibits cell migration via regulating actin polymerization and focal adhesions yet induces capillary tube formation and angiogenesis via binding SH3 protein interacting with Nck (SPIN90/WISH) in endothelial cells [142] (figure 2).

In the tumour microenvironment, IGPR-1 expressed on the cell surface of endothelia binds HHLA2, expressed not only on the tumour cells but also on tumour-associated macrophages. These paracrine signalling interactions aid tumour progression, resulting in immunosuppressed tumour milieu and angiogenic stimulation [145,146]. Further, colon cancer cells also upregulate IGPR-1 expression to enhance tumour cell–cell interaction, or ‘multicellular aggregation’, in order to optimize survival and protect against chemotherapeutic agents [147]. Recently, it was shown that NEDD4 ubiquitin E3 ligase binds to IGPR-1 and mediates its polyubiquitination leading to lysosomal degradation [148], suggesting that inhibitors of the lysosomal pathway could enhance IGPR-1 levels. Notably, both nutrient deprivation and amino acid starvation induce IGPR-1 activation through Ser220 phosphorylation. In turn, activated IGPR-1 induces phosphorylating activation of AMPK, thus stimulating canonical autophagic machinery downstream, including Unc-51-like kinase 1 (ULK1), Beclin 1, and lipidated microtubule-associated protein light chain 3 (LC3-II) [149] (figure 2). Collectively, these observations bridge the gap between cell adhesion and autophagy, positing IGPR-1 as a novel pro-autophagic and pro-angiogenic cell surface receptor at the endothelial-cancer cell interface.

## 5. Anti-autophagic signalling

**Figure 3.**
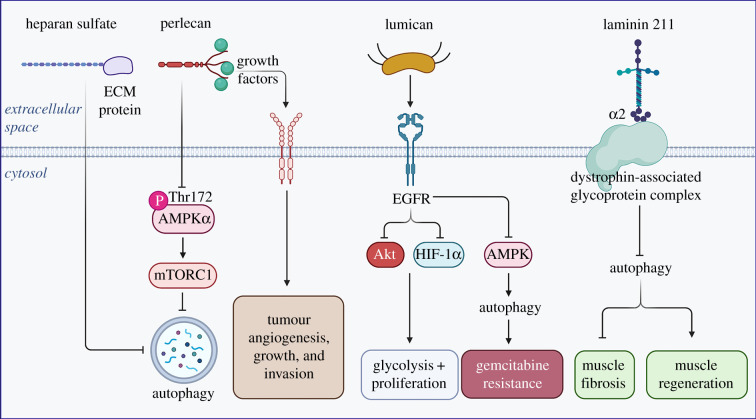
Schematic depiction of various anti-autophagic ECM components, their respective receptors and key downstream signalling events. Please consult the text for additional information. The graphic was created with BioRender.com.

One of the largest matrix proteins with a protein core of approximately 470 kDa, perlecan is an important HSPG deposited extracellularly in basement membranes, cartilage, bone marrow and muscle [150,151]. Perlecan regulates a broad spectrum of biological processes, not limited to angiogenesis [152–158], autophagy [151,159], endocytosis [160], cell adhesion [161,162], thrombosis [163], blood-brain barrier integrity [164] and lipid catabolism [165,166]. By contrast with its pro-autophagic C-terminal domain V endorepellin, perlecan as a whole inhibits autophagy via mTOR complex 1 (mTORC1) activation (figure 3). Mice lacking perlecan expression through *Hspg2* deletion showed increased autophagy due to inadequate mTORC1 activation in their slow-twitch soleus muscles, as demonstrated by elevated levels of autophagic markers LC3-II and phosphorylated AMPK alongside decreased phosphorylation of p70S6 K, a downstream target of mTORC1 [167].

As part of its pro-angiogenic mechanism of action, perlecan binds growth factors through its heparan sulfate chains and presents them to their cognate receptors [168,169]. These growth factors include a number of angiokines such as VEGFA, PDGF, FGFs 2, 7 and 18 and progranulin [46,170–173]. In line with its ability to bind and facilitate growth factor signalling, perlecan in the tumour matrix promotes tumour angiogenesis, growth and invasion [55,157,172,174,175]. Further, its expression is significantly elevated in metastatic melanoma and other cancer cell lines [51,153,176,177]. CAFs in pancreatic cancer also secrete high levels of perlecan into the tumour microenvironment to aid in metastasis [178].

Yet another small leucine-rich proteoglycan, lumican, regulates a host of physiological and disease processes, including fibrocyte differentiation [179], wound healing [180,181], glucose homeostasis [182], inflammation [183,184], cell migration [185], cancer [186–188] and angiogenesis [189]. In cancer, lumican responds in a tissue-specific manner, both inhibiting cancer proliferation in melanoma [190,191] and pancreatic [192,193], breast [194–196] and prostate cancers [197] while inducing growth and metastasis in lung [198] and gastric cancers [199,200]. In pancreatic ductal adenocarcinoma (PDAC) following surgical resection, high levels of lumican are correlated with decreased recurrence and prolonged patient survival, implicating lumican as an anti-oncogenic agent in this tissue-specific context. Further, lumican signals as an EGFR antagonist in PDAC, thereby blocking Akt signalling and HIF-1α activity downstream and suppressing glycolysis and proliferation [192,193]. Importantly, lumican inhibits autophagy in PDAC via downregulating AMPK activity (figure 3). Therefore, since autophagy confers resistance against the chemotherapeutic agent gemcitabine in PDAC, extracellular lumican effectively sensitizes cancer cells to chemotherapy treatment via autophagic inhibition [201].

Laminin α2 is a subunit of heterotrimeric laminin 211, a prominent laminin isoform found in the basement membranes of skeletal muscle, Schwann cells and astrocytes and pericytes of brain capillaries [202]. Laminins function as scaffold proteins attaching the cell surface to the ECM. For instance, the carboxyl end of laminin α2 binds to the dystrophin-associated glycoprotein complex, whereas the amino end binds both α1β1 and α2β1 integrins [203]. Dysfunctional laminin α2 caused by mutations within the *LAMA2* gene encoding laminin α2 results in *LAMA2-*related muscular dystrophies (MDC1A), the most common subtype of congenital muscular dystrophy [204]. Laminin α2 itself is an autophagy inhibitor, as autophagy genes and proteins are upregulated in laminin α2-deficient muscle in an MDC1A mouse model (figure 3). Notably, systemic pharmacological inhibition of autophagy reduced muscle fibrosis and increased muscle regeneration and mass, resulting in improving overall life expectancy and locomotion [205]. Additional studies elucidating the mechanism between autophagy and muscle homeostasis and regeneration is needed and would expand our understanding of autophagy and its biological impact in tissue systems. In cancer biology, laminin α2 also plays diverse roles in cancer. For example, laminin 211 binds to integrin α7 to effectively stimulate the proliferation of acute myeloid leukaemia cells with granulocytic sarcoma via increased ERK signalling [206]. Separately, *LAMA2* expression inhibits lung adenocarcinoma metastasis, such that destabilizing *LAMA2* mRNA through Mex3a binding promotes lung adenocarcinoma cell migration and invasion [207]. In glioblastoma (GBM), laminin α2 promotes GBM cell growth and self-renewal and is correlated to negative patient prognoses [208]. Finally, laminin α2 expression is upregulated in the basement membranes of endothelial cells supplying lung small and large cell neuroendocrine carcinomas, resulting in metastasis through an EGF-dependent pathway [209]. These studies further elucidate the cancer-specific role of laminin α2 in cell migration and proliferation.

As cellular growth is often linked to heparan sulfate-carrying proteoglycans, it is not surprising that HSPGs functioning as co-receptors to promote growth factor activity and positioned within signalling networks could also be involved in regulating autophagy (figure 3). In *Drosophila*, downregulating heparan sulfate biosynthesis increases autophagy at the neuromuscular junction [210]. Diminishing heparan sulfate levels globally activates autophagy-dependent processes, increasing lifespan, reactive oxygen species (ROS) resistance and decreases build-up of ubiquitin-modified proteins following ROS exposure [211]. Of interest is the observation that low molecular weight heparin, an over-sulfated form of heparan sulfate, prevents autophagic induction in activated neutrophils and the formation of neutrophil extracellular traps [212]. Another glycosaminoglycan, chondroitin sulfate, binds and activates protein tyrosine phosphatase receptor sigma (PTPRσ) which, in turn, dephosphorylates cortactin [213], a PTPRσ-interacting protein identified by proximity-labelling assay [214]. This inhibitory activity occurs at the autophagosome-lysosome fusion step thereby disrupting autophagy flux at axonal tips and ultimately leading to axonal growth inhibition [213]. The sulfation pattern of the glycosaminoglycan chain determines the precise glycan length that interacts with PTPRσ and defines the fate of axonal regeneration through a complex interaction among PTPRσ, cortactin and autophagy [215]. These studies are in agreement with the well-known effect of chondroitinase ABC in promoting axonal growth and functional recovery after spinal cord injury [216]. Overall, these findings implicate heparan and chondroitin sulfate as effective inhibitors of autophagy. The mechanism by which these two glycosaminoglycans inhibit autophagy has yet to be fully elucidated. How these extracellular polysaccharides influence intracellular recycling and degradation and the receptors and signalling molecules at play is an interesting question, and its answer would benefit both the glycobiology and autophagy fields.

## 6. The field is expanding: heparanase and thrombospondin 1

The field linking autophagy to ECM has been recently extended to include heparanase, an endoglucuronidase that uniquely cleaves cell surface heparan sulfate chains, basement membranes and ECM proteoglycans [217]. Mechanistically, heparanase releases a plethora of growth factors, cytokines and chemokines bound to heparan sulfate chains in the glycocalyx and stromal counterparts, thereby playing a vital role in creating a permissive environment for cell growth [218–221]. As part of a complex network of remodelling enzymes [222], heparanase is known to promote myeloma stemness and tumorigenesis [223] and synergize with chemotherapy to drive macrophage activation and cancer growth [224]. Furthermore, anti-myeloma chemotherapy evokes secretion of exosomes enriched in heparanase that remodel the tumour stroma and contribute to chemoresistance and patient relapse [225]. Notably, targeting heparanase to the mammary epithelium promotes tumour growth and metastasis [226]. Mice transplanted with bone marrow-derived from heparanase-overexpressing transgenic mice show enhanced tumour aggressiveness and shorter survival times [227]. Moreover, heparanase enhances autophagy, and this process favours tumour growth and chemoresistance [228–230]. Clinical trials testing heparanase inhibitors as an anti-cancer therapy show early signs of efficacy [231], further underscoring the clinical importance of this enzyme [218].

An intriguing ECM constituent is the matricellular protein thrombospondin 1 (Thbs1), one of the first anti-angiogenic factors to be identified and studied [232]. Thbs1 binds with high affinity to CD47, a ubiquitously expressed cell surface receptor which mediates global cardiovascular function and responses to stress [233]. Notably, CD47 deficiency acts as a pro-survival factor through activation of the autophagic flux; thus, CD47 blockade could act as a modulator of autophagy and radioprotection [234]. These observations have been expanded to invasive breast cancer where a combinatorial treatment of anthracyclines and anti-CD47 antibodies inhibits cancer growth while preventing cardiac toxicity via autophagy induction [235]. More recent studies have shown that CD47 mediates autophagy in RAS-expressing cancer cell lines and triggers tumour growth inhibition [236]. In agreement with these studies, intact CD47 signalling suppresses autophagy following renal IRI in both native and transplanted kidneys, in contrast to *CD47^−/−^* kidneys which exhibit a robust increase in autophagic markers [237]. Thus, targeting CD47 in acute renal injury could ameliorate of renal function following injury [237]. Finally, it is worth noting that the anti-angiogenic Thbs1 has been recently identified as an autophagy activator by triggering the PERK–eF2α–ATF4 stress axis [238], similar to that evoked by anti-angiogenic endorepellin in endothelial cells [239]. Specifically, Thbs1, but not Thbs2–4, induces lethal cardiac atrophy via stress-evoked autophagy and acts as a critical regulator of cardiomyocyte size in the stressed heart [238]. Obviously, some of these recent reports need to be independently confirmed in different experimental models and human pathologies by other investigators. Nonetheless, a central theme that is emerging is that disturbances in matrix constituents and molecules with high affinity for ECM affect the intracellular process of self-eating, and ultimately the outcome of cancer and other diseases.

## 7. Conclusion and future perspectives

A growing body of research reveals the impact of autophagy on ECM function. For example, the realization that autophagy plays a key role in intervertebral disc and cartilage biology [240] and wound healing [241] provides some hints for future directions and a prospective translational impact for therapeutic application. Another intriguing observation is the link between syndecan 4, a member of the cell surface HSPGs, and autophagy as it relates to alveolar bone resorption in periodontitis [242]. Yet another recent observation is the report that progranulin, a growth factor that interacts with perlecan [172], is a negative regulator of autophagy [243]. The playing field of proteoglycan research is expanding, given the discovery of novel proteoglycans using mass spectrometry [244] in *Caenorhabditis elegans*, and glycoproteomics approaches in both *C. elegans* and mammalian cells [245–247]. Intriguingly, some of these novel chondroitin sulfate proteoglycans are canonical prohormones, typically stored and secreted from granules of endocrine cells [248].

Although ECM-driven processes that drive cancer are profound [2], the vast majority of cancer research is restricted to the cancer cell itself. As a result, less focus is placed on ECM pathology within the tumorigenic milieu. However, in effort to curtail cancer growth on all fronts, it is equally important to address the deleterious effects on tumorigenesis stemming from the ECM as much as it is to address the metastatic and proliferative changes in the cancer cell. Importantly, research focused on how a dysfunctional ECM fundamentally drives disease processes such as desmoplasia, EMT and tumour vascularization is fundamental in treating cancer from an extracellular front. Within this realm of ECM-driven pathology in cancer, an emerging body of work demonstrates that autophagic modulators are important in perpetuating oncogenesis.

Another recent intriguing observation is that the tissue/substrate stiffness can modulate stroma cell metabolism in an AMPK-dependent mechanism. Specifically, matrix stiffness alone is sufficient to evoke autophagy in stromal fibroblasts, enabling them to create a pro-oncogenic niche supporting neighbouring cancer cells [249].

However, several outstanding questions remain. Given the stage-dependent role of autophagy in tumorigenesis, at what stage are these pro- and anti-autophagic proteoglycans playing a role in either suppressing or promoting tumour development? Are the downstream effects of these autophagic modulators occurring separately or do they coordinate synergistically in the tumour microenvironment? Are certain proteoglycans more critical in modulating disease-altering autophagy in certain cancers over others? Thus, understanding the interplay of anti- and pro-autophagic matrix molecules in aberrant matrix remodelling and their respective functions in tumorigenesis should not be overlooked. Overall, further investigation into these inquiries is necessary to better understand the disease-driving impact of the ECM in carcinogenesis.

## Data accessibility

This article has no additional data.
